# Aetiological distribution of pulmonary hypertension and the value of transthoracic echocardiography screening in the respiratory department: A retrospective analysis from China

**DOI:** 10.1111/crj.13623

**Published:** 2023-05-04

**Authors:** Cheng Hong, Riken Chen, Linna Hu, Haimin Liu, Jianmin Lu, Chunying Zhuang, Wenliang Guo, Xiaofeng Wu, Jielong Lin, Xishi Sun, Haiyang Tang, Zhewen Wang, Nuofu Zhang, Zhenzhen Zheng

**Affiliations:** ^1^ Guangzhou Medical University, State Key Laboratory of Respiratory Disease, National Clinical Research Centre for Respiratory Disease, Guangzhou Institute of Respiratory Health The First Affiliated Hospital of Guangzhou Medical University Guangzhou Guangdong China; ^2^ Graduate School Guangzhou University of Chinese Medicine Guangzhou Guangdong China; ^3^ The Second Affiliated Hospital of Guangdong Medical University Zhanjiang Guangdong China; ^4^ Department of Respiratory and Critical Care Medicine The Second Affiliated Hospital of Guangdong Medical University Zhanjiang Guangdong China

**Keywords:** pulmonary hypertension, respiratory department, right heart catheterization, transthoracic echocardiography

## Abstract

**Methods:**

The aetiological composition and clinical characteristics of patients with pulmonary hypertension (PH) hospitalised in the respiratory department were retrospectively analysed, as well as the correlation between transthoracic echocardiography (TTE) and right heart catheterization (RHC) for evaluating pulmonary artery systolic pressure (PASP) and mean pulmonary artery pressure (mPAP).

**Results:**

Of 731 patients, 544 (74.42%) were diagnosed with PH by RHC. Pulmonary arterial hypertension (PAH) was the most common type of PH, accounting for 30.10%; PH due to lung disease and/or hypoxia accounted for 20.79%, and PH due to pulmonary artery obstructions accounted for 19.29%. TTE has the highest specificity for diagnosing PH due to pulmonary artery obstructions. The specificity was 0.9375, the sensitivity was 0.7361 and the area under the ROC curve (AUC) was 0.836. PASP, and mPAP estimated by TTE were different for various types of PH. In terms of PASP, TTE overestimated PASP in PH due to lung disease and/or hypoxia, but there was no significant difference compared with RHC (P > 0.05). TTE underestimates PAH patients' PASP compared with RHC. In terms of mPAP, TTE underestimated the mPAP of all types of PH, as there was a significant difference in the TTE‐estimated mPAP of patients with PAH compared with RHC but not on other types of PH. Pearson correlation analysis of TTE and RHC showed a moderate overall correlation (rPASP 0.598, P < 0.001; rmPAP 0.588, P < 0.001).

**Conclusions:**

Among the patients with PH in the respiratory department, patients with PAH accounted for the majority. TTE has high sensitivity and specificity for the diagnosis of PH due to pulmonary artery obstructions in the respiratory department.

## Graphical abstract

This study confirmed that the distribution of PH aetiology in respiratory department differs from the total population. TTE is more suitable for the diagnosis of pulmonary artery obstructive pulmonary hypertension in the respiratory department, but overall, there are still limitations compared to RHC and further studies are needed to set the threshold values.

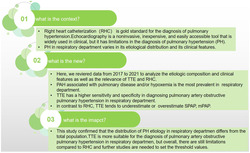

## 1 INTRODUCTION

Pulmonary hypertension (PH) presents as an idiopathic form of complications associated with circulatory disease characterised by increased pulmonary vascular resistance (PVR), increased pulmonary artery blood flow and elevated left cardiac filling pressure that can lead to cardiac overload and right ventricular (RV) dysfunction.^1^ PH is caused by various lung, heart or other systemic diseases.^2^, ^3^ The clinical classification of PH includes approximately 50 different pathological conditions divided into five different groups, all of which have a common detectable elevated pulmonary artery pressure (PAP). The five groups of PH are as follows: (1) pulmonary artery hypertension (PAH), a group of PH characterised haemodynamically by the presence of pre‐capillary PH, defined by an mPAP ≥ 25 mmHg, pulmonary artery wedge pressure (PAWP) ≤ 15 mmHg and a PVR > 3 Wood units (WU) in the absence of other causes of pre‐capillary PH such as PH due to lung diseases, chronic thromboembolic pulmonary hypertension (CTEPH) or other rare diseases, mainly includes idiopathic pulmonary artery hypertension (IPAH), heritable pulmonary arterial hypertension (HPAH), drug‐ or toxin‐associated PAH and disease‐associated PAH; (2) PH due to left heart disease, which is defined by an mPAP ≥ 25 mmHg and a PAWP > 15 mmHg. Within this haemodynamic condition of post‐capillary PH, IpcPH is defined by PVR ≤ 2 WU and combined postcapillary and precapillary pulmonary hypertension (CpcPH) by PVR > 2 WU; (3) PH due to lung disease and/or hypoxia; the most common lung diseases associated with PH are chronic obstructive pulmonary disease (COPD), interstitial lung disease and combined pulmonary fibrosis and emphysema (CPFE); (4) PH due to CTEPH and/or other pulmonary artery obstructions, which is a disease of obstructive PA remodelling as a consequence of major vessel thromboembolism and (5) PH with an unclear and/or multifactorial mechanism, includes several disorders with multiple patho‐aetiologies.^1^ PH is a hidden malignant disease with a high rate of missed diagnosis and misdiagnosis and is associated with poor prognosis. N‐terminal pro B type natriuretic peptide (NT‐proBNP) and endothelin‐1, as markers of PH, have been used to evaluate the condition and prognosis of patients in recent years, but their sensitivity and specificity are not satisfactory. During the pathogenesis of PH, due to diagnostic level limitations, patients often miss the best period of treatment. In the respiratory department, the examination of a series of biochemical indicators does not often attract clinicians' attention, especially when symptoms such as chest tightness, shortness of breath or dyspnoea are so similar to the symptoms of many respiratory diseases, which inevitably require time for the investigation of causes. Patients with severe PH and symptoms such as right heart failure and lower limb oedema are often delayed to a later stage. Therefore, early detection and treatment are of vital importance for patients with PH.

The gold standard for the diagnosis of PH is right heart catheterization (RHC). Although RHC is safe,^4^ it is an invasive procedure that is not suitable for wide application. As the primary screening method for PH, transthoracic echocardiography (TTE) is recommended as the first‐tier method for diagnosing PH, evaluating cardiac haemodynamics, monitoring patient condition, determining prognostic factors, and evaluating treatment outcomes. TTE is also a safe method extensively used to evaluate the structure and function of the RV in patients with PH. However, the accuracy of TTE for measuring PAP remains elusive.^5^, ^6^, ^7^ The respiratory department is one of the first departments in which patients with PH are referred to. Summarising the aetiological composition and clinical characteristics of patients with PH in the respiratory department can effectively improve clinicians' understanding of PH. This study described 731 patients with suspected PH admitted to the Department of Respiratory and Critical Care Medicine of our hospital (National Respiratory Medicine Centre) from January 2015 to January 2021. The aetiological composition and clinical characteristics of the patients were analysed, and the correlation between TTE and RHC for estimating haemodynamic parameters was evaluated, as well as the diagnostic efficacy of TTE in patients with various kinds of PH.

By analysing the aetiological composition and clinical characteristics of PH in the respiratory department, we can improve clinicians' understanding of PH. We aimed to evaluate the correlation between pulmonary artery pressure (PAP) obtained by TTE and PAP obtained by right heart catheterization (RHC) and to determine the diagnostic accuracy of TTE for PH.

## 2 MATERIALS AND METHODS

### 2.1 General data

A total of 731 hospitalised patients with suspected PH admitted to the National Centre for Respiratory Medicine from January 2015 to January 2021 were confirmed through RHC conducted by the same group according to the same SOP. The diagnostic criteria for PH were based on the 2015 ESC/ERS Guidelines for the Diagnosis and Treatment of Pulmonary Hypertension.^1^ In the resting state, the mean pulmonary arterial pressure (mPAP) measured by RHC was ≥25 mmHg. Among the patients, there were 370 females and 361 males, with an age of 52.17 ± 16.84 years old and a body mass index (BMI) of 21.38 ± 4.17 kg/m^2^.

### 2.2 Methods

PH was defined as mPAP ≥ 25 mmHg (1 mmHg = 0.133 kPa) at rest as measured by RHC. The clinical data of patients with PH included sex, age, symptoms, signs, biochemical and immune biomarkers, blood gas analysis, pulmonary function test, chest computed tomography (CT), ventilation‐perfusion (V/Q) lung scan and other imaging data. Patients with incomplete data or unclear diagnoses were excluded after biochemical and immune biomarkers, pulmonary function and V/Q scans were reverified for the aetiological diagnosis of PH by the researchers. RHC was performed to record haemodynamic data, including mPAP, mean right atrium pressure (mRAP), PVR, pulmonary artery wedge pressure (PAWP), cardiac index (CI) and so forth. After the patients were classified according to their specific PH type, correlation analysis and group comparisons were performed with the indicators collected above.

The examination of TTE was performed by professional doctors in the echocardiogram room. The instrument was a GE Vivid E9 colour Doppler ultrasound diagnostic instrument with an M5s probe and probe frequency of 1.7–3.4 MHz. The examinees were in the left lateral decubitus position, breathing calmly and synchronously connected to the ECG. Examinees underwent echocardiography, during which the right atrial inner diameter (two‐dimensional ultrasound measured the short axis inner diameter and transverse diameter in the middle of the right atrium at the end of systolic phase in the four‐chamber apical section), right ventricular inner diameter (two‐dimensional ultrasound measured the transverse diameter of the late diastolic right ventricular base segment in the four‐chamber apical section), Tricuspid Annular Plane Systolic Excursion (TAPSE), velocity of tricuspid annular systolic phase (S'), ratio of early and late peak velocity of diastolic tricuspid orifice (E/A), peak velocity of early diastolic tricuspid movement (E/E'), right myocardial performance index (RMPI), and Pulmonary Artery Systolic Pressure (PASP) were measured and calculated. RMPI was calculated from the right ventricular isovolumic relaxation, isovolumic contraction and ejection time measured by tissue Doppler. According to the following formula: (isovolumetric dilation + isovolumetric systole)/ejection phase time, an RMPI>0.55 indicates right ventricular insufficiency. The most common method for estimating PASP is continuous wave Doppler measurement of the right ventriculus‐right atrial pressure gradient reflected in the peak velocity of tricuspid regurgitation, which is then added to the modified Bernoulli equation, as follows: PASP (mmHg) = 4 × TRV2 + RAP, with the estimated right atrial pressure.^8^ The parameters and methods of measurement were in accordance with the Guidelines for the echocardiographic assessment of the right heart in adults.^9^ mPAP was derived from the equation (mPAP = 0.61 × PASP+2) proposed by Chemla et al.^8^

RHC was performed in the catheterization laboratory within 24 h of TTE by two experienced operators (X.W. and C.H.) who accessed the internal jugular or femoral vein under fluoroscopic guidance using a liquid‐filled Swan‐Ganz 7‐Fr catheter. All RHC studies were performed with local anaesthesia at the puncture site. The RAP, pulmonary artery diastolic pressure (PADP), pulmonary artery systolic pressure (PASP), mPAP, and PCWP were recorded at the end of normal expiration. Cardiac output (CO, L/min) was determined with the thermodilution method and the Fick method using a direct measure of oxygen uptake. Finally, PVR (WU) was calculated by using the following formula: PVR = [(mPAP−PCWP)/CO].

Several measurements were taken, and averages were calculated over five cardiac cycles in the case of atrial fibrillation (AF) and/or supraventricular or ventricular premature beats and over three cardiac cycles in the case of sinus rhythm.

All procedures carried out in this study were consistent with the ethical standards of the Institutional Research Council and the 1964 Declaration of Helsinki and subsequent amendments or similar ethical standards. All the individual participants included in the study provided written informed consent. The protocol was approved by the local ethics committee (ethics approval number 2020135).

#### 2.2.1 Statistical analysis

The SPSS 22.0 statistical analysis software was used for the statistical analysis. Clinical data are described as the mean ± standard deviation, and Pearson correlation analysis was used for correlation analysis. A receiver operating characteristic (ROC) curve was drawn to evaluate the diagnostic efficiency of TTE. Analysis of variance (ANOVA) was used to compare the data between multiple groups, the Z test was used to compare the area under each curve, and the chi‐square test was used to compare the rates between groups. P < 0.05 was considered statistically significant.

## 3 RESULTS

### 3.1 Clinical baseline data

**FIGURE 1:**
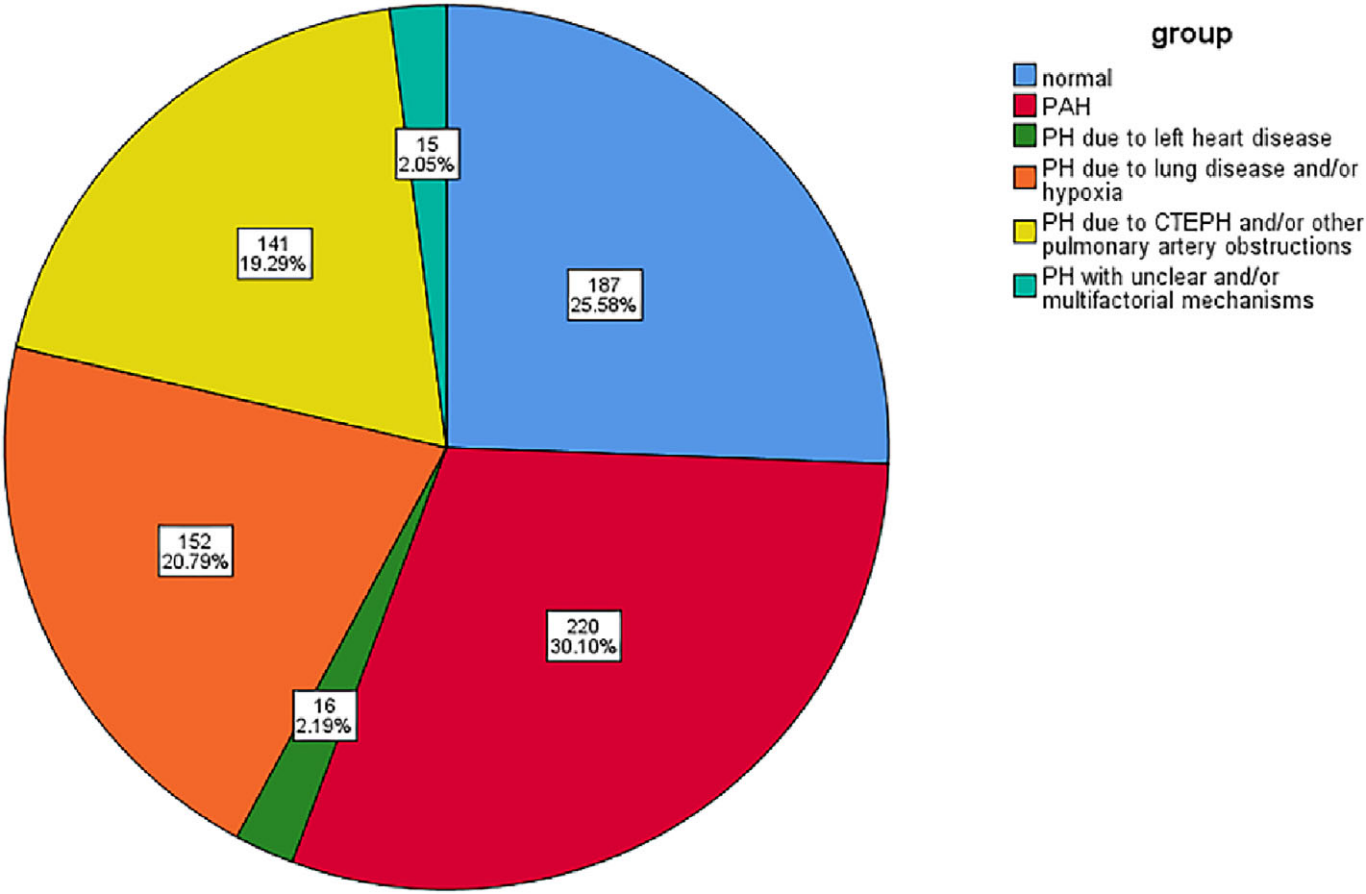
Population distribution of pulmonary hypertension.

A total of 731 hospitalised patients (average age 52.17 ± 16.84 years; 370 female) with suspected PH were admitted to the National Centre of Respiratory Medicine from January 2015 to January 2021, and all underwent RHC examination. Table 1 shows the baseline clinical data of the main subjects, including sex, age, BMI, blood pressure, heart rate, lung function, proBNP, serum creatinine, uric acid, thyroid stimulating hormone, thyroid hormone and so forth. The results showed that most patients with PH due to lung disease and/or hypoxia had decreased pulmonary ventilation function, mainly obstructive ventilation function disturbance. Pro‐BNP was higher than other types of PH in patients with PAH. Ultrasonographic Doppler parameters evaluated by routine TTE showed moderate elevation of PASP in most patients. Left ventricular systolic function decreased in patients with PH due to left heart disease. At the same time, the right atrial, left atrial and left ventricular end‐diastolic diameters were enlarged, whereas left ventricular systolic function was mostly normal in patients with other types of PH. Of the 731 patients with haemodynamic parameters measured by RHC, 544 (74.42%) were diagnosed with PH by RHC. The correlation population distribution (Figure 1) showed that 187 patients had normal mPAP, accounting for 25.58%, whereas 544 patients were confirmed as having PH, with a female: male ratio of 1.15. The total composition ratio of the five PH groups was as follows: (1) PAH in 220 cases (30.10%), among which females accounted for 67.70%; (2) PH due to left heart disease was found in 16 cases (2.19%); (3) PH due to lung disease and/or hypoxia in 152 cases (20.79%); (4) PH due to CTEPH and/or other pulmonary artery obstructions in 141 cases (19.29%); and (5) PH with unclear and/or multifactorial mechanisms was found in 15 cases (2.05%).

**TABLE 1:** Characteristics of the patients at baseline.

Characteristic	Normal	All patients	I	II	III	IV	V	P
No. (%)	187 (25.58)	731 (10)	220 (30.10)	16 (2.19)	152 (20.79)	141 (19.29)	15 (2.05)	‐
Female sex no. (%)	79 (42.20)	370 (50.62)	149 (67.70)	7 (43.80)	43 (28.30)	88 (62.40)	4 (26.70)	<0.001
Age, year	55.71 ± 13.97	52.17 ± 16.84	41.43 ± 18.99	61.31 ± 13.79	57.46 ± 13.54	56.45 ± 13.71	51.21 ± 10.75	<0.001
BMI, kg/m^2^	21.83 ± 4.86	21.38 ± 4.17	21.25 ± 3.94	23.92 ± 3.55	20.50 ± 4.36	21.86 ± 3.50	19.84 ± 2.02	0.137
SBP, mmHg	126.41 ± 20.44	122.24 ± 21.18	114.64 ± 20.10	123.50 ± 22.64	126.08 ± 21.83	122.61 ± 19.82	125.00 ± 24.76	<0.001
DBP, mmHg	77.79 ± 13.53	76.03 ± 12.73	73.45 ± 12.16	77.08 ± 6.96	77.38 ± 12.85	75.42 ± 12.10	78.27 ± 13.74	<0.05
mBP, mmHg	93.90 ± 14.82	91.00 ± 15.17	86.26 ± 14.63	94.25 ± 13.28	93.16 ± 14.55	90.80 ± 15.72	94.00 ± 16.07	<0.001
HR, bpm	85.68 ± 16.08	86.09 ± 16.24	84.90 ± 16.73	94.00 ± 20.21	90.88 ± 16.70	81.98 ± 13.79	88.93 ± 12.34	<0.001
PASP, mmHg^a^	28.02 ± 7.73	58.91 ± 29.38	80.20 ± 26.66	54.15 ± 16.35	51.37 ± 14.82	74.45 ± 25.74	67.33 ± 28.40	<0.001
PADP, mmHg^a^	10.09 ± 4.63	24.28 ± 13.88	34.44 ± 13.95	27.31 ± 8.90	23.79 ± 7.43	26.89 ± 12.04	26.87 ± 13.28	<0.001
mPAP, mmHg^a^	17.37 ± 4.95	37.59 ± 18.29	51.47 ± 16.63	39.13 ± 12.98	35.47 ± 9.88	44.30 ± 14.84	42.80 ± 18.36	<0.001
PAWP, mmHg^a^	5.89 ± 3.81	8.65 ± 6.84	8.42 ± 6.55	18.19 ± 9.53	10.69 ± 9.17	8.88 ± 4.56	12.07 ± 8.58	<0.001
PVR, WU^a^	2.39 ± 1.12	7.04 ± 5.80	10.48 ± 6.30	5.00 ± 3.61	5.62 ± 3.54	10.54 ± 6.04	5.85 ± 4.31	<0.001
CO, L/min^a^	5.34 ± 1.47	4.77 ± 1.67	4.40 ± 1.74	4.55 ± 1.44	5.07 ± 1.64	3.99 ± 1.40	5.83 ± 2.07	<0.001
CI, L/min/m^2^ ^**a**^	3.37 ± 0.96	3.14 ± 1.43	3.17 ± 2.13	2.86 ± 0.91	3.27 ± 0.98	2.57 ± 0.93	3.46 ± 1.33	<0.05
PASP, mmHg^b^	32.19 ± 11.65	54.85 ± 30.84	67.63 ± 32.78	51.31 ± 26.32	53.22 ± 25.70	66.09 ± 33.41	64.60 ± 33.39	<0.001
mPAP, mmHg^b^	21.31 ± 6.99	34.91 ± 18.51	42.58 ± 19.67	32.79 ± 15.79	33.93 ± 15.42	41.65 ± 20.04	40.76 ± 20.03	<0.001
RA, mm^b^	31.80 ± 11.93	35.34 ± 18.05	40.80 ± 19.46	40.00 ± 17.07	31.80 ± 17.15	35.10 ± 20.42	30.00 ± 18.34	<0.001
EF, %^b^	67.08 ± 12.05	64.88 ± 16.22	66.31 ± 15.43	45.40 ± 26.65	63.70 ± 17.47	64.12 ± 17.46	61.07 ± 15.21	<0.001
LA, mm^b^	29.46 ± 7.24	28.93 ± 6.81	28.36 ± 7.82	41.44 ± 10.86	27.79 ± 5.54	29.13 ± 4.45	27.67 ± 4.59	<0.001
LVDd, mm^b^	42.82 ± 7.09	40.16 ± 9.25	37.75 ± 9.76	53.80 ± 19.35	40.27 ± 9.00	38.33 ± 7.23	43.67 ± 7.17	<0.001
FEV1, %	65.07 ± 30.49	66.95 ± 28.72	75.80 ± 20.20	62.28 ± 47.31	45.82 ± 25.03	85.46 ± 21.31	77.22 ± 16.25	<0.001
FEV1/FVC, %	73.29 ± 20.12	72.11 ± 18.46	77.30 ± 10.26	69.00 ± 24.55	64.45 ± 24.61	72.50 ± 11.20	67.34 ± 11.50	<0.001
proBNP, pg/mL	177.13 ± 367.25	991.09 ± 2210.19	1743.90 ± 3220.24	1512.90 ± 1289.22	1058.78 ± 1919.97	938.12 ± 2019.31	1423.22 ± 1864.69	0.001
SCr, μmoI/L	78.38 ± 27.28	75.81 ± 29.04	72.54 ± 29.46	80.75 ± 44.09	73.05 ± 30.95	77.60 ± 23.65	103.15 ± 38.26	<0.05
UA, μmol/L	367.90 ± 144.12	409.31 ± 155.51	437.75 ± 156.40	523.80 ± 237.09	367.25 ± 137.49	460.82 ± 158.93	438.83 ± 118.90	0.001
TSH, μU/mL	2.18 ± 2.96	2.37 ± 2.56	3.01 ± 2.93	1.82 ± 1.20	1.78 ± 1.21	2.42 ± 2.62	2.48 ± 1.60	<0.05
FT4, pmol/L	12.56 ± 3.57	13.30 ± 4.44	13.80 ± 5.84	13.04 ± 3.21	13.34 ± 3.59	13.13 ± 2.96	16.31 ± 9.29	0.123
T4, nmol/L	110.08 ± 26.35	110.99 ± 27.65	112.97 ± 30.60	115.17 ± 21.91	108.41 ± 25.63	110.17 ± 22.94	124.83 ± 56.20	0.611

WHO functional class								
I	3 (7.9%)	20 (6.1%)	8 (6.0%)	0 (0.0%)	4 (7.1%)	5 (6.3%)	0 (0.0%)	
II	23 (60.5%)	130 (39.5%)	53 (39.8%)	3 (23.1%)	14 (25.0%)	33 (41.3%)	4 (44.4%)	
III	10 (26.3%)	155 (47.1%)	62 (46.6%)	7 (53.8%)	33 (58.9%)	38 (47.5%)	5 (55.6%)	
IV	2 (5.3%)	24 (7.3%)	10 (7.5%)	3 (23.1%)	5 (8.9%)	4 (5.0%)	0 (0.0%)	

*Note*: Plus‐minus values are means ± SD. I = PAH, II = PH due to left heart disease, III = PH due to lung disease and/or hypoxia, IV = PH due to CTEPH and/or other pulmonary artery obstructions and V = PH with unclear and/or multifactorial mechanisms.

Abbreviations: BMI, body mass index; CI, cardiac index; CO, cardiac output; DBP, diastolic blood pressure; EF, ejection fraction; FEV1, forced expiratory volume; FT4, serum free thyroxine; FVC, forced vital capacity; HR, heart rate; LA, left atrium; LVDd, left ventricular end‐diastolic dimension; mBP, mean blood pressure; mPAP, mean pulmonary arterial pressure; PADP, pulmonary artery diastolic pressure; PASP, pulmonary artery systolic pressure; PAWP, pulmonary artery wedge pressure; proBNP, pro B type natriuretic peptide; PVR, pulmonary vascular resistance; RA, transverse meridian of right atrium; SBP, systolic blood pressure; SCr, serum creatinine; T4, thyroid hormone; TSH, thyroid stimulating hormone; UA, uric acid.

^a^ Values were measured by right heart catheterization.

^b^ Values were measured by cardiac ultrasound.

#### 3.1.1 ROC curve was used to analyse the sensitivity and specificity of TTE in the diagnosis of PH in the respiratory department

**FIGURE 2:**
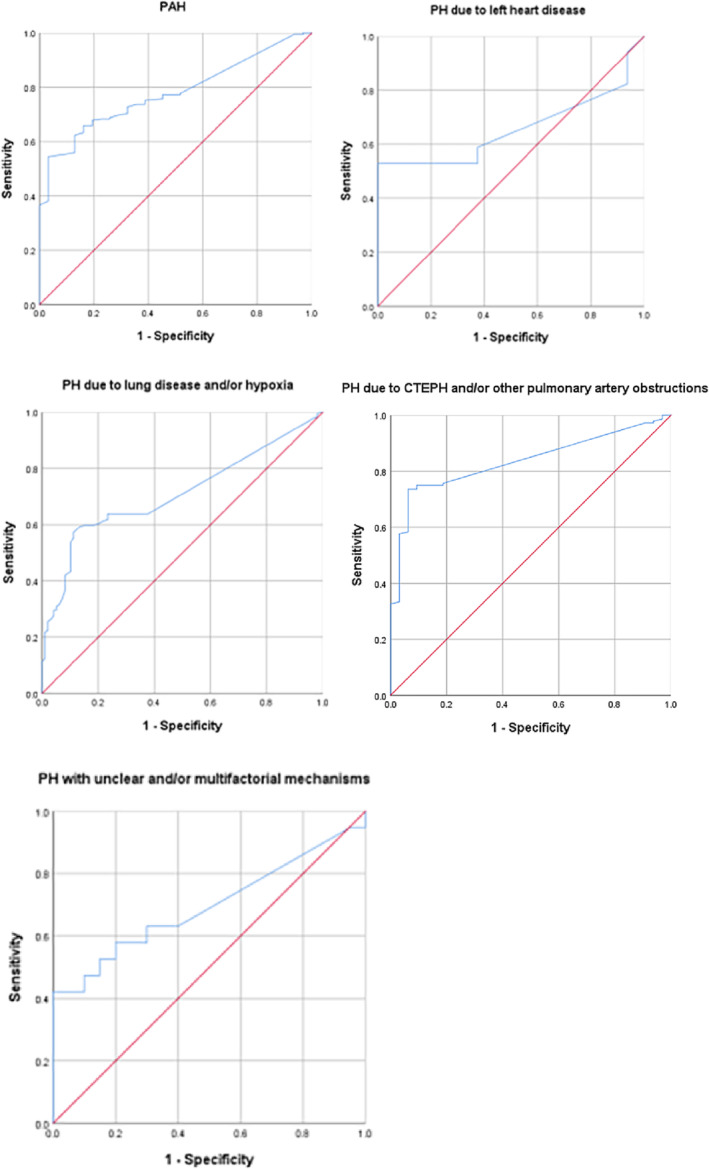
Receiver operating characteristic (ROC) curve of five types of pulmonary hypertension. The above is the ROC curve analysis of five types of pulmonary hypertension patients.

(1) There were 233 PAH patients (31.87%) with a sensitivity of 0.7723 (95% confidence interval [CI] 0.7145–0.8301), a specificity of 0.5484 (95% CI 0.3732–0.7236) and a weighted area under the ROC curve (AUC) of 0.778 (95% CI 0.711–0.845, P < 0.001); (2) 33 patients (4.51%) with PH due to left heart disease had a sensitivity of 0.5294 (95% CI 0.2921–0.7667), specificity of 0.7500 (95% CI 0.5378–0.9622) and an AUC of 0.656 (95% CI 0.456–0.856, P = 0.126); (3) 250 patients (34.20%) with PH due to lung disease and/or hypoxia showed a sensitivity of 0.6316 (95% CI 0.5549–0.7083), a specificity of 0.7653 (95% CI 0.6814–0.8492) and an AUC of 0.710 (95% CI 0.647–0.774, P < 0.001); (4) PH due to CTEPH and/or other pulmonary artery obstructions were found in 176 patients (24.08%), with a sensitivity of 0.7361 (95% CI 0.6641–0.8081), a specificity of 0.9375 (95% CI 0.8536–1.0214) and an AUC of 0.836 (95% CI 0.772–0.899, P < 0. 001); and (5) 39 patients (5.34%) with PH due to unclear and/or multifactorial mechanisms had a sensitivity of 0.5790 (95% CI 0.3569–0.8010), a specificity of 0.8000 (95% CI 0.6247–0.9731) and an AUC of 0.695 (95% CI 0.522–0.868, P < 0.05) (Table 2, Figure 2).

**TABLE 2:** ROC curve analysis of five types of pulmonary hypertension evaluated by cardiac ultrasound.

Group	AUC (95% CI)	P	Youden index	Sensitivity (95% CI)	Specificity (95% CI)	PPV	NPV	DOR
I	0.778 (0.711–0.845)	<0.001*	0.5123	0.7723 (0.7145–0.8301)	0.5484 (0.3732–0.7236)	0.9177 (0.8763–0.9590)	0.2698 (0.1602–0.3795)	4.12
II	0.656 (0.456–0.856)	0.126	0.5294	0.5294 (0.2921–0.7667)	0.7500 (0.5378–0.9622)	0.6923 (0.4414–0.9432)	0.6000 (0.3853–0.8147)	3.38
III	0.710 (0.647–0.774)	<0.001*	0.4601	0.6316 (0.5549–0.7083)	0.7653 (0.6814–0.8492)	0.8067 (0.7358–0.8777)	0.5725 (0.4878–0.6572)	5.59
IV	0.836 (0.772–0.899)	<0.001*	0.6667	0.7361 (0.6641–0.8081)	0.9375 (0.8536–1.0214)	0.9815 (0.9561–1.0069)	0.4412 (0.3232–0.5592)	41.84
V	0.695 (0.522–0.868)	<0.05*	0.4211	0.5790 (0.3569–0.8010)	0.8000 (0.6247–0.9753)	0.7333 (0.5095–0.9571)	0.6667 (0.4781–0.8553)	5.50

Abbreviations: AUC, area under the ROC curve; CI, confidence interval; DOR, diagnostic odds ratio; NPV, negative predictive value; PPV, positive predictive value; ROC, receiver operating characteristic.

* *p* < 0.05.

#### 3.1.2 Analysis of the difference and correlation between TTE and RHC in the estimation of PAP

The values of PASP and mPAP estimated by TTE were lower than those estimated by RHC, and the difference was statistically significant (P < 0.001) (Tables 3 and 4). In PASP and mPAP estimation, the correlation between TTE and RHC was moderate (rPASP = 0.598, P < 0.001; rmPAP = 0.588, P < 0.001) (Tables 3 and 4).

**TABLE 3:** Analysis of the difference and correlation between PASP^a^ and PASP^b^.

Group	PASP^a^	PASP^b^	p	r	p
Normal	28.02 ± 7.73	32.19 ± 11.65	<0.001*	0.121	0.098
All patients	58.91 ± 29.38	54.85 ± 30.84	<0.001*	0.598	<0.001*
I	80.20 ± 26.66	67.63 ± 32.78	<0.001*	0.361	<0.001*
II	54.15 ± 16.35	51.31 ± 26.32	>0.05	0.665	<0.05*
III	51.37 ± 14.82	53.22 ± 25.70	>0.05	0.486	<0.001*
IV	74.45 ± 25.74	66.09 ± 33.41	0.001	0.530	<0.001*
V	67.33 ± 28.40	64.60 ± 33.39	>0.05	0.713	<0.05*

Abbreviation: PASP, pulmonary artery systolic pressure.

^a^ Values were measured by right heart catheterization.

^b^ Values were measured by cardiac ultrasound.

* *p* < 0.05.

**TABLE 4:** Analysis of the difference and correlation between mPAP^a^ and mPAP.^b^

Group	mPAP^a^	mPAP^b^	p	r	p
Normal	17.37 ± 4.95	21.31 ± 6.99	<0.001*	‐0.002	>0.05
All patients	37.59 ± 18.29	34.91 ± 18.51	<0.001*	0.588	<0.001*
I	51.47 ± 16.63	42.58 ± 19.67	<0.001*	0.338	<0.001*
II	39.13 ± 12.98	32.79 ± 15.79	>0.05	0.586	<0.05*
III	35.47 ± 9.88	33.93 ± 15.42	>0.05	0.481	<0.001*
IV	44.30 ± 14.84	41.65 ± 20.04	>0.05	0.551	<0.001*
V	42.80 ± 18.36	40.76 ± 20.03	>0.05	0.699	<0.05*

Abbreviation: mPAP, mean pulmonary artery pressure.

^a^ Values were measured by right heart catheterization.

^b^ Values were measured by cardiac ultrasound.

* *p* < 0.05.

#### 3.1.3 Difference and correlation analysis of estimated PAP by TTE and RHC in the respiratory department

(1) PASP and mPAP estimated by TTE in a normal population were higher than those estimated by RHC, and the difference was statistically significant (P < 0.001). Pearson correlation analysis between TTE and RHC showed no statistical significance (rPASP = 0.121, P = 0.098; rmPAP = 0.002, P > 0.05) (Tables 3 and 4). (2) PASP and mPAP estimated by TTE in PAH were lower than those estimated by RHC, and the difference was not statistically significant (P > 0.05). There was a moderate correlation between the two measures (rPASP = 0.665, P < 0.05; rmPAP = 0.586, P < 0.05) (Tables 3 and 4). (3) The PASP and mPAP of PH due to left heart disease estimated by TTE were lower than those estimated by RHC, and the difference was not statistically significant (P > 0.05). The data from two measurements showed moderate correlation (rPASP = 0.665, P < 0.05; rmPAP = 0.586, P < 0.05) (Tables 3 and 4). (4) The PASP estimated by TTE for pulmonary disease and/or hypoxemia‐associated PH was higher than that estimated by RHC, and the mPAP estimated by RHC was lower than that estimated by TTE, with no statistical significance (P > 0.05). These two measurements showed a low correlation (rPASP = 0.486, P < 0.001; rmPAP = 0.481, P < 0.001) (Tables 3 and 4). (5) The PASP estimated by TTE in PH due to CTEPH and/or other pulmonary obstructive lesions was lower than that estimated by RHC, the difference was statistically significant (P = 0.001) and the mPAP estimated by TTE was lower than that estimated by RHC, with no statistical significance (P > 0.05) (Tables 3 and 4). The two measurements were moderately correlated (rPASP = 0.530, P < 0.001; rmPAP = 0.551, P < 0.001) (Tables 3 and 4 and Tables 5 and 6 in Data S1) For PH with unclear and/or multifactorial mechanisms, PASP and mPAP estimated by TTE were lower than those estimated by RHC, and the difference was not statistically significant (P > 0.05) (Tables 3 and 4). Correlation analysis showed that there was a moderate correlation between TTE and RHC in PASP estimation (rPASP = 0.713, P < 0.001) and mPAP estimation (rmPAP = 0.699, P < 0.001) (Tables 3 and 4).

## 4 DISCUSSION

The epidemiological description of PH in the 2015 ESC guidelines reveals^1^ that Group 2 (PH due to LHD) has the highest prevalence, although the exact figure is not known. The prevalence of PAH is higher than that of CTEPH, whereas the prevalence of other groups is not reported. It seems that there are relatively more epidemiological data for PAH and CTEPH, whereas there are relatively less epidemiological data for other groups of PH. A systematic review^10^ of the literature evaluating recent (5‐year) national systematic registry data from centralised health care systems found that the incidence of PAH was approximately 5.8 cases per million, the prevalence of PAH was approximately 47.6–54.7 cases per million, the incidence of CTEPH was approximately 3.1–6.0 cases per million, and the prevalence of CTEPH was approximately 25.8–38.4 cases per million. As a national clinical and research institute of respiratory diseases in China, we registered 731 patients with suspected PH for RHC and 566 patients were diagnosed with PH, the ratio of female to male ratio is 1.15. The higher prevalence in women is consistent with the description in the guidelines.^1^ In our study, the lower percentage of respiratory PH patients was found in groups 2 (PH due to LHD) and 5 with 16 and 15 patients, respectively. The low number of patients in group 2 is different from the highest prevalence about this group mentioned in the guidelines.^1^ The mainly reason is that most patients who consulted in the respiratory department are mainly with respiratory diseases; moreover, physicians can identify patients who have PH due to LHD based on their clinical experience and the patient's clinical presentation then usually referred them to the cardiovascular department. The other three groups with the highest to lowest percentage of aetiological distribution in our study were PAH, PH due to lung diseases and/or hypoxaemia and CTEPH. The higher prevalence of PAH than CTEPH is consistent with the guidelines and other literature reports.^1^, ^10^, ^11^ In our study, group 3 patients were 20.79% of the enrolment, which is close to the results of a US PH surveillance survey^12^ by Hyduk et al. As seen in our study, the aetiological distribution of PH in respiratory medicine consists mainly of PH due to PAH, pulmonary disease and/or hypoxemia and CTEPH; PAH (group1) is the most common cause of PH in the respiratory department.

In our study, we found that the range of mPAP values varied in different groups of PH in the respiratory department. The mean value of mPAP measured by RHC was 37.59 ± 18.295 in patients with PH in the respiratory department, which is moderate. The mPAP in PAP is severe, with a mean value of 51.47 ± 16.63 mmHg, and mPAP in other groups of PH being moderate (36‐45 mmHg). Among them, the mPAP of PAH and CTEPH were close to the mPAP of the US as well as the Spanish PH registry.^11^, ^13^ The mPAP of the third group of PH registered in our centre was lower than the other groups, which is 35.47 ± 9.88 mmHg. Among them, forced expiratory volume (FEV1) and FEV1/forced vital capacity (FVC) were significantly lower than those of patients in other PH groups, 45.82% ± 25.03% and 64.45% ± 24.61%, respectively. Consequently, it can be observed that the third group of patients had mainly moderate to severe obstructive ventilation dysfunction and were predominantly COPD (FEV1/FVC < 70%) with a Global Initiative for Chronic Obstructive Lung Disease (GOLD) classification of stage 3. Previous reports in the literature suggested^14^, ^15^, ^16^ that PH elevation was mainly found in patients with GOLD stage 4 and that mPAP was mild, generally less than 30 mmHg, but in our study, we found that PH elevation was mainly found in patients with COPD GOLD stage 3, and mPAP was moderate; therefore, we need to pay attention not only to GOLD stage 4 but also to patients with GOLD stage 3. Chaouat et al^17^ found a mismatch between pulmonary function and PH in a subset of COPD patients, which we will continue to focus on in later studies.

RHC is the gold standard for the diagnosis of PH, and TTE has become a common method for the assessment of PH because of its advantages of non‐invasiveness, economy and accessibility; TTE has also been recommended by many guidelines as the first choice of examination for patients with suspected PH.^1^ The diagnosis of PH is based on mPAP measured by RHC. The mean pulmonary artery pressure process of echocardiography^18^ can be calculated using the application of the equation (MPAP = 0.6 × SPAP + 2) proposed by Chemla.^8^ Aduen et al^19^ compared the retrograde RV and RA pressure gradient methods as well as the Chemla equation for calculating mPAP, and the results suggested that the two methods were equally applicable to clinical practice.

Ni et al^20^ conducted a meta‐analysis of 27 studies and found that the overall sensitivity and specificity of TTE were 85% and 74%, respectively; the positive likelihood ratio was 3.2; the negative likelihood ratio was 0.20; the diagnostic odds ratio (DOR) was 16, and the AUC was 0.88. This study found that TTE had different sensitivities and specificities in the diagnosis of PH in the respiratory department. We found that TTE had good sensitivity and specificity in the diagnosis of CTEPH, with an overall sensitivity of 0.7361, specificity of 0.9375, positive likelihood ratio of 0.9815, negative likelihood ratio of 0.4412 and diagnostic odds ratio DOR of 41.84.Therefore, TTE is more appropriate for the diagnosis of CTEPH. PAH had the highest sensitivity at 77%; however, the specificity was low at 54%. Previous literature has reported^18^ much lower diagnostic accuracy of echocardiography in patients with PH associated with respiratory disease. Anatomical factors (especially hyperinflation) make it impossible to estimate RVSP in most patients, and even if RVSP can be estimated, it is usually inaccurate and usually differs from RHC measurements by more than 10 to 20 mmHg. A study by Arcasoy et al^21^ also found that optimal visualisation of the heart was compromised in COPD patients due to hyperventilation, resulting in only 44% of patients could have PASP measurements by TTE, whereas 52% of PASP pressure value estimates were found to be inaccurate (>10 mmHg difference compared with the pressure obtained during RHC). Unlike previous studies, we observed that the sensitivity and specificity of PH due to lung disease and/or hypoxia (n = 250) were moderate, 0.6316 and 0.7653, respectively; whereas, the difference with RHC measurements was less than 5 mmHg.

In our study, the sensitivity, specificity and AUC of TTE in the diagnosis of PH due to left heart disease were 0.5294, 0.7500 and 0.656, respectively. Compared with other types of PH, TTE seems have a relatively poor predictive value for PH due to left heart disease (Table 2). Interestingly, we found that the majority of respiratory patients with PH due to left heart disease had decreased FEV1 and FEV1/FVC, implying that these patients often have chronic airway diseases, such as COPD, in combination. Arrhythmias or loss of atrioventricular synchronisation and often combine with the chronic airway disease may have affected the validity of TTE in measuring PH in this group of patients; the specific reasons need to be further explored.

The difference analysis of PASP and mPAP estimated by TTE and RHC showed that PASP and mPAP estimated by TTE were generally lower than those estimated by RHC, and the difference was significant (P < 0.001) (Tables 3 and 4). Fisher et al^22^ found that TTE could overestimate or underestimate PASP, and the frequency of overestimation and underestimation was similar (16 cases and 15 cases, respectively); whereas, the range of underestimation was greater than that of overestimation. Another prospective study suggested that TTE may often overestimate PAP.^6^ This study found that TTE‐estimated PAH in the respiratory department varies among different populations and different types of PH. TTE overestimated PAP in the normal population, and the difference was significant compared with RHC. TTE generally underestimated PASP and mPAP in RHC‐confirmed PH (544 cases, 74.42%). The differences in TTE‐estimated PASP and mPAP varied among different types of PH. TTE overestimated PASP in patients with pulmonary disease and/or hypoxemia‐associated PH, but there was no significant difference between TTE and RHC. The PASP of PAH would be underestimated by TTE, and the difference was significant compared with RHC. Although TTE underestimated the PASP of patients with other types of PH, there was no significant difference. Different from previous studies, our study found that TTE tends to underestimate mPAP, but without a significant difference, except for that of PAH, in which the difference was significant compared with RHC (Tables 3 and 4).

A prospective study by Stephane et al^22^ found a strong linear correlation between PASP and mPAP (mPAP = 0.6 × PASP + 2, r = 0.97, P < 0.0001). Rich et al^6^ compared PASP estimated by TTE and PASP measured by an invasive method in 183 patients with PH and found that there was a moderate correlation between the TTE and RHC measurements of PASP. The results of Andrea et al^23^ also showed that there was a moderate correlation between PASP and mPAP as assessed by TTE and RHC, with r values of 0.65 and 0.60, respectively. The results of a meta‐analysis^24^ showed that the correlation between TTE‐ and RHC‐estimated PASP ranged from r = 0.65 (P < 0.001) to r = 0.97 (P < 0.001). The comprehensive sensitivity, specificity and accuracy of TTE for the diagnosis of PH were 88% (95% CI 84–92%), 56% (95% CI 46–66%) and 63% (95% CI 53–73%), respectively.

**FIGURE 3:**
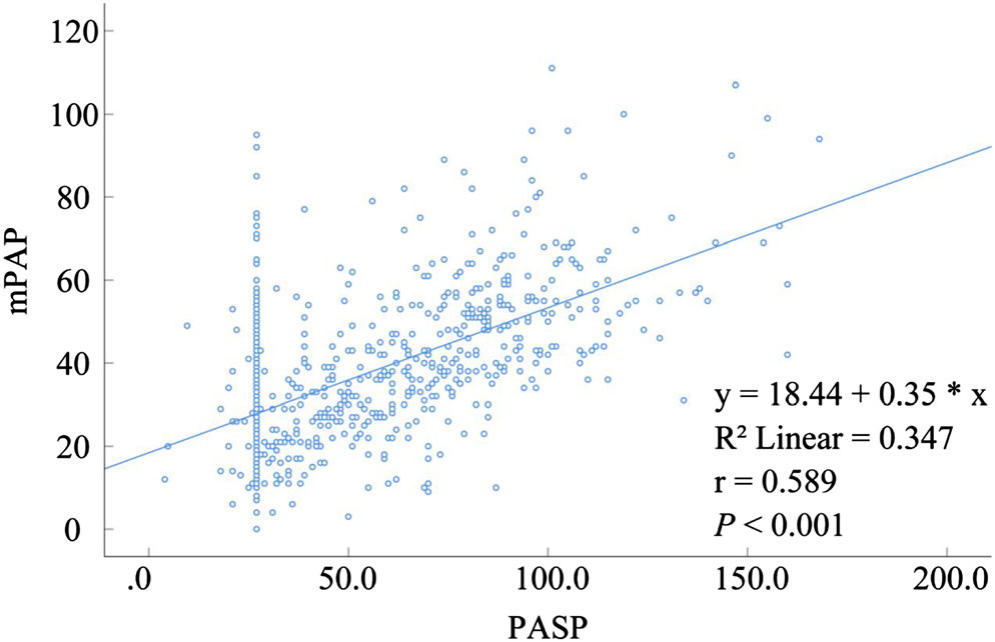
Scatter plot with PASP ↨ as abscissa and mPAP ∩ as ordinate in PH.

In this study, Pearson correlation analysis between TTE and RHC showed a moderate overall correlation (rPASP = 0.598, P < 0.001; rmPAP = 0.588, P < 0.001) (Figure 3; Tables 3 and 4). Correlation analysis of TTE‐ and RHC‐measured mPAP and PASP were different for different types of PH in the respiratory department. The normal population's correlation between PASP and mPAP estimated by TTE showed no statistical significance (Tables 3 and 4). Finkelhor et al^25^ also found that TTE correlated poorly with RHC in patients with normal PASP. Because TTE overestimates PASP and mPAP values in the normal population, relevant guidelines^9^ also recommend an estimate of PASP of >35 to 40 mmHg, followed by RHC to determine the presence of PH. In patients with PAH, the correlation between TTE‐ and RHC‐estimated PASP and mPAP values were weak, and the specificity of TTE for diagnosing patients with PAH was low as mentioned earlier. The correlation between TTE and RHC in PH due to left heart disease and PH due to CTEPH and/or other pulmonary obstructive lesions was moderate. TTE estimated that PASP and mPAP were highly correlated with RHC in patients with unexplained pulmonary hypertension, and the sensitivity of TTE for diagnosing this group of patients was 57.9%; the specificity was 80%, and the accuracy was 69.5%. Therefore, the sensitivity and accuracy of TTE for the estimation of patients with unexplained pulmonary hypertension were relatively low, but because the SPAP and mPAP of these patients were highly correlated with RHC, it may be used as a tool to monitor SPAP and mPAP after the diagnosis of these patients with PH.

## 5 CONCLUSION

This study showed that PAH, PH due to lung disease and/or hypoxia and PH due to CTEPH and/or other pulmonary artery obstructions were the main causes of PH in the respiratory department. TTE has a high specificity and sensitivity in the evaluation of PH due to CTEPH and/or other pulmonary artery obstructions, so it can be used as a means to diagnose and evaluate this type of PH. TTE is undoubtedly an effective tool for screening, aetiology analysis and prognosis assessment. It can provide structural and functional information about the heart that cannot be obtained by RHC. However, as a diagnostic tool, echocardiography still has some limitations, and the effect of different types of PH on TTE examination varies. Further prospective studies are needed to set different cut‐off values for different types of PH to further strengthen the value of diagnosis an assessment of the disease.

## AUTHOR CONTRIBUTIONS

All authors contributed to the writing, review and approval of the final copy of the manuscript.

## CONFLICT OF INTEREST STATEMENT

None declared.

## ETHICS STATEMENT

The protocol was approved by the local ethics committee (ethics approval number: 2020135).

## Supporting information


**Data S1.** Supporting Information

## Data Availability

The authors confirm that the data supporting the findings of this study are available within the article.
