# Supplementary material for: Aetiological distribution of pulmonary hypertension and the value of transthoracic echocardiography screening in the respiratory department: A retrospective analysis from China
Source: Clin Respir J. 2023 May 4;17(6):536–47. doi: 10.1111/crj.13623 (PMC10265155; doi:10.1111/crj.13623)

Table 5. Comparison of the correlation between mPAP **∩** and various indexes in group N, group A,5 Types of Pulmonary Hypertension.

| group | mPAP ∩ -PASP ↨ | mPAP **∩** -PAWP **∩** | mPAP ∩-EF ↨ | mPAP **∩** -proBNP |
| --- | --- | --- | --- | --- |
|  | r p | r p | r p | r p |
| normal | -0.002 0.977 | 0.279 <0.001^*^ | 0.091 0.254 | 0.045 0.684 |
| All patients | 0.589 <0.001* | 0.166 <0.001^*^ | 0.079 0.043 | 0.308 <0.001^*^ |
| I | 0.338 <0.001* | 0.058 0.410 | 0.180 <0.05^*^ | 0.153 0.175 |
| II | 0.586 <0.05* | 0.461 0.083 | 0.235 0.398 | -0.775 0.123 |
| III | 0.481 <0.001* | 0.079 0.344 | 0.104 0.213 | 0.204 0.124 |
| IV | 0.551 <0.001* | 0.006 0.945 | 0.144 0.100 | 0.297 0.056 |
| V | 0.699 <0.05* | 0.348 0.204 | 0.305 0.268 | 0.205 0.659 |

* p < 0.05

Table 6. Comparison of the correlation between PASP ↨ and various indexes in group N, group A,5 Types of Pulmonary Hypertension.

| group | PASP ↨-PAWP **∩** | PASP ↨- EF | PASP ↨-proBNP | PASP ↨- PASP **∩** |
| --- | --- | --- | --- | --- |
|  | r p | r p | r p | r p |
| normal | -0.100 0.190 | -0.128 0.109 | -0.115 0.302 | 0.121 0.098 |
| All patients | 0.013 0.729 | 0.056 0.150 | 0.305 <0.001^*^ | 0.598 <0.001^*^ |
| I | -0.076 0.282 | 0.096 0.190 | 0.161 0.155 | 0.361 <0.001^*^ |
| II | 0.286 0.302 | 0.629 <0.05^*^ | 0.909 <0.05^*^ | 0.665 <0.05^*^ |
| III | -0.125 0.133 | 0.066 0.429 | 0257 0.052 | 0.486 <0.001^*^ |
| IV | -0.085 0.336 | 0.100 0.252 | 0.482 =0.001^*^ | 0.530 <0.001^*^ |
| V | 0.257 0.354 | -0.172 0.540 | 0.430 0.335 | 0.713 <0.05^*^ |

^*^ p < 0.05

**Figure 3.**Scatter plot with PASP ↨as abscissa and mPAP ∩as ordinate in PH .


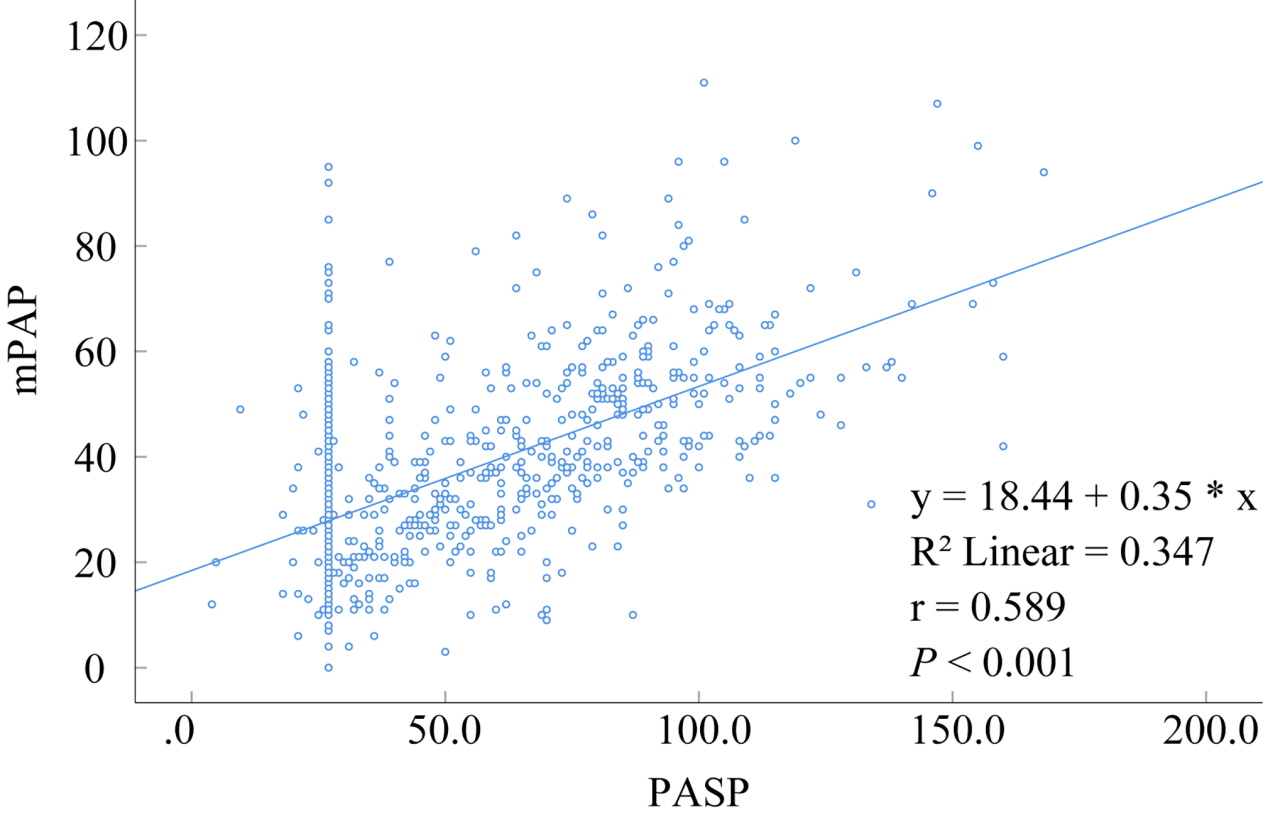

Supplement: Supplementary file 1 — Data S1. Supporting Information [file CRJ-17-536-s001.docx]
